# Sacrificial Reducing Agent Free Photo-Generation of Platinum Nano Particle over Carbon/TiO_2_ for Highly Efficient Oxygen Reduction Reaction

**DOI:** 10.1038/srep37006

**Published:** 2016-11-15

**Authors:** Rajashekar Badam, Raman Vedarajan, Kazuki Okaya, Koichi Matsutani, Noriyoshi Matsumi

**Affiliations:** 1 School of Materials Science, Japan Advanced Institute of Science and Technology, 1-1 Asahidai, Nomi, Ishikawa 923-1292, Japan; 2 Tanaka Kikinzoku Kogyo K.K, Nagatoro 2-14, Hiratsuka, Kanagawa 254-0021, Japan

## Abstract

Electrocatalytic materials for oxygen reduction reaction, currently dominated by platinum/carbon catalyst is marred by drawbacks such as use of copious amount of Pt and use of “non-green” sacrificial reducing agent (SRA) during its synthesis. A single stroke remedy for these two problems has been achieved through an *in-situ* aqueous photoreduction void of even trace amounts of SRA with an enhanced activity. Reduction of PtCl_6_^2−^ salt to Pt nano particles on carbon substrate was achieved solely using solar spectrum as the source of energy and TiO_2_ as photocatalyst. Here, we demonstrate that this new procedure of photoreduction, decorates Pt over different types of conducting allotropes with the distribution and the particle size primarily depending on the conductivity of the allotrope. The Pt/C/TiO_2_ composite unveiled an ORR activity on par to the most efficient Pt based electrocatalyst prepared through the conventional sacrificial reducing agent aided preparation methods.

The rise in greenhouse gases and the depletion of fossil fuels lead the researchers to look for efficient alternative energy conversion and storage devices. Two major flagship devices in energy conversion and storage technologies are fuel cell and Li-air battery respectively. For complete realization of these technologies, the following interdependent challenges have to be addressed, sluggish kinetics of oxygen reduction reaction (ORR), non-green methods for catalyst production and high cost of catalyst preparation. Though there has been many attempts to make non-platinum based ORR catalyst, Pt has been a universal choice due to its efficient and reliable electro-catalytic activity. In general, Pt based carbon catalysts, one of the most widely employed ORR catalyst, are prepared by employing polyols and/or other reducing agents and are subjected to elevated temperatures. The use of these procedures stray outside the “green-way”. Few researchers have showed surfactant free but pyrolytic methods of ORR catalyst preparation on carbon nitride based substrates to reduce the cost of the catalyst^1^,^2^. Lookout for alternative green procedures has recently ended up in looking into exploiting photo-reduction processes. Alongside ‘green’ aspect, large scale commercial applications are precluded by the high manufacturing cost of the ORR catalyst. Hence, researcher have been focusing on reducing the cost of the catalyst by reducing the amount of Pt used by adapting to a variety of carbon materials, use of transition metal oxides and doping of nitrogen in carbon. The year 2015, has seen more developments and attempts to improve the ORR activity by the addition of a metal oxide such as MoO_x_^3^, TiO_x_^4^ and CeO_x_^5^,^6^, on the Pt/C catalyst. Incidentally some or most of these metal oxides fall in the category of photo-catalyst. Generation of charges on impingement of light, characteristic to any photo-catalyst, can potentially involve in various photo-physical phenomenon like trapping of charges, radiative and non-radiative charge recombination along with interfacial charge transfer. The interfacial charges are available for further redox reactions of the molecules adsorbed onto the surface of the photo-catalyst.

In this regard, TiO_2_ nanostructures have been studied in-depth and have proven to be an excellent choice for photocatalytic reduction of many materials ranging from fullerenes^7^, inorganic^8^ and many organic compounds^9^. Here, the high conducting band of TiO_2_ (approx. −0.5 V vs NHE at 7.00 pH) enables controlled reduction of chemical species, which is highly necessary. This validates the thermodynamic feasibility to transfer the photo-generated charges especially electrons to any conducting carbon for further reduction of metal salts^10^. In the recent past among many strategies to control the recombination of electron–hole, carbon-TiO_2_ hybrid are more looked upon due to its efficiency^11^,^12^,^13^,^14^,^15^,^16^,^17^,^18^,^19^. The control of recombination in TiO_2_/C hybrid is achieved owing to the placement of the conduction band of carbon marginally lower than that of TiO_2_ allowing a predominant passage of electrons from conduction band of TiO_2_. This process of interfacial charge transfer to the pre-adsorbed conducting carbons, was found to be a working alternative to control the rapid recombination of the photo-generated charges of TiO_2_. This spill-over of electrons from the conduction band, when happens on a highly conducting matrix, the electrons tend to travel over the conducting matrix until it finds a suitable reducing species. Recently few researchers^20^,^21^,^22^ tapped these photo-electrons for recovering few transition metals from industrial wastes in aqueous media without any added SRA. Yunteng *et al*.^23^ and Ian *et al*.^24^ contributed significantly in photo-reducing the metals salts to metal nanoparticles on to carbon substrates. However, they added a determined amount of SRA and it was not straight a forward reaction to scale them up industrially. Along with the novelty in the method, some of the recent works^23^,^25^,^26^ have reported that Pt-carbon-TiO_2_ three component system shows enhanced electro-catalytic activity with high durability.

In this article we present a novel and facile photochemical reaction tapping the photo-electron generated by semiconductor when irradiated with simulated solar light. The photo-electron is successfully tapped onto carbon which further reduces chloroplatinic acid to nano particle of high electrocatalytic activity. The whole process is carried out without any SRA, pH adjustment or temperature variation in water. To the best of our knowledge this article will be the first of its kind highlighting successful photo-reduction of metal salts to metal nano particles on to any electron conducting substrate using TiO_2_ nano particles as photo catalysts. The three component system thus prepared, exhibited promising ORR activity with ultralow amount of Pt capable to replace ORR catalysts with high Pt content.

## Results and Discussion

**Figure 1:**
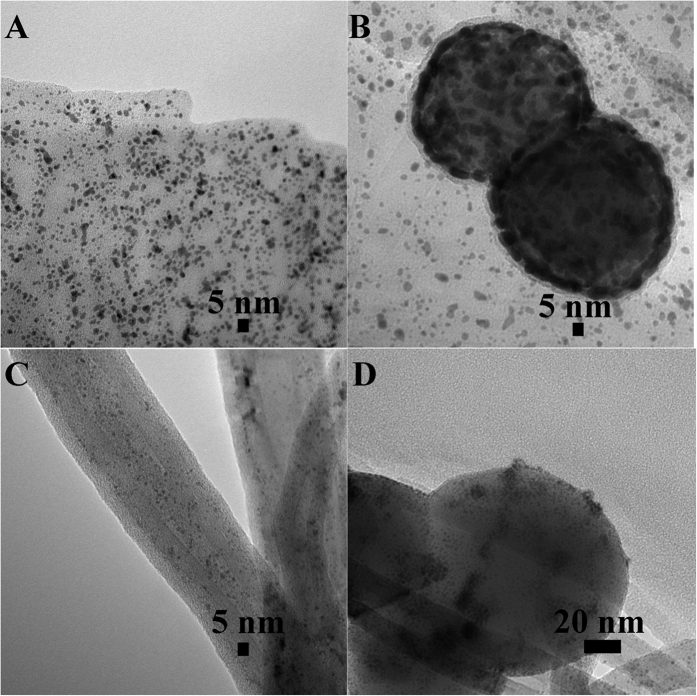
Morphology. TEM micrographs of Photo-Pt-graphite-TiO_2_ (**A,B**) Photo-Pt-CNT-TiO_2_ (**C,D**).

The exact quantity of Pt loading on each sample quantified using (inductively coupled plasma mass spectrometry) ICP-MS was found to be 4.3, 1.6 and 2.6 wt% in Photo-Pt-Graphite-TiO_2_, Photo-Pt-GO-TiO_2_ and Photo-Pt-CNT-TiO_2_, respectively. The TEM images obtained for the carbon/TiO_2_ surfaces after subjecting to photo-irradiation was observed to possess dark, particle-like contrast. Similar textural aspect was observed on both CNT as well as graphite/TiO_2_ hybrids. Figure 1A,B shows the TEM micrographs of both the materials (Photo-Pt-graphite-TiO_2_ and Photo-Pt-CNT-TiO_2_) made using photo-reduction method. Closely looking at Fig. 1A, uniformly distributed particles were seen over graphite sheet. Figure 1B shows a dark spot of ~50 nm, correlating to the structure of TiO_2_ on a 2-dimensional sheet of graphite, which was seemingly embedded over vast span of dark minute particulate embodies. Closer look reveals that the TiO_2_ particle is evidently covered by the dark particle. The particles are uniformly distributed without any agglomerations all through the graphite. The average size of the particles were 2.3 ± 1.5 nm on graphite and 5–8 nm on TiO_2_. Similar results were observed on CNT i.e., Photo-Pt-CNT-TiO_2_ (Fig. 1C) and the average size of the particle over CNT was observed to be 1 ± 03 nm and 2–4 nm on TiO_2_ (Fig. 1D). As a spin-off, similar methodology was adapted with Graphene Oxide (GO), in case of GO, when TiO_2_ was used as photo-catalyst the particles decorated only the TiO_2_ (Figure ESI 1) with the size ranging around 2–6 nm and not on GO.

**Figure 2:**
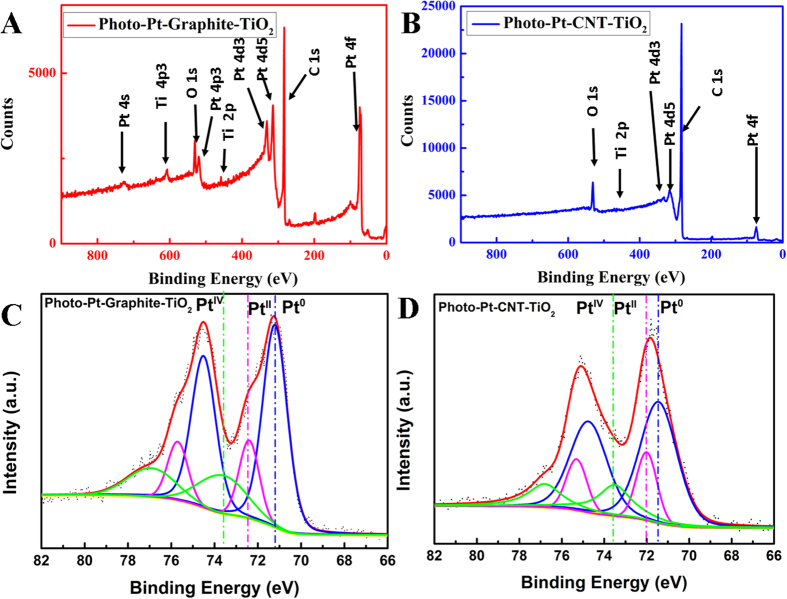
Chemical composition of Pt: Shows high resolution XPS spectra of Pt4f. of Photo-Pt-Graphite-TiO_2_ and Photo-Pt-CNT-TiO_2_.

**Figure 3:**
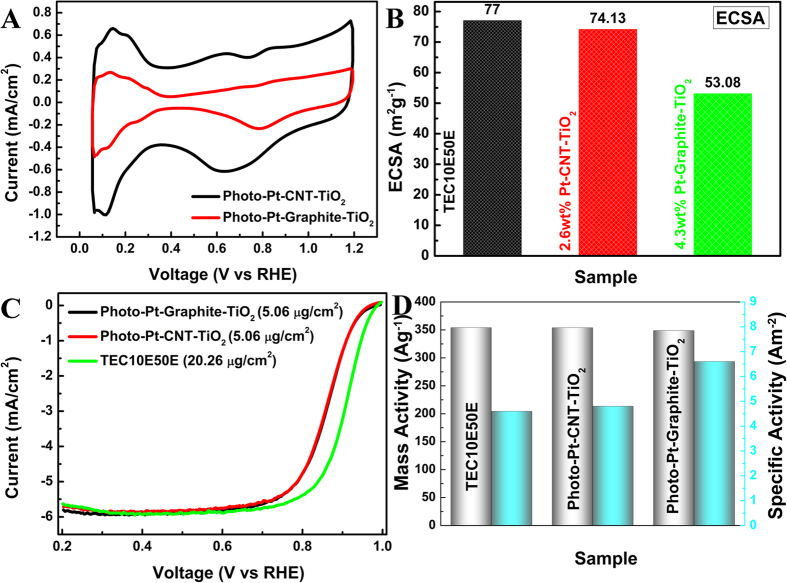
ORR characteristics of electrocatalysts. (**A**) CV of Photo-Pt-Graphite-TiO_2_ and Photo-Pt-CNT-TiO_2_ in N_2_ saturated 0.1 M HClO_4_ aq. at scan rate of 50 mVs^−1^. (**B**) ECSA calculated from the CVs and comparison with commercial material. (**C**) RDE linear sweep voltammograms for all the samples under study and (**D**) Mass activity and Specific activity obtained from K-L plots at 0.90 V vs RHE.

The elemental composition of the electrocatalytic materials prepared was determined by XPS. Figure 2A,B are the typical survey spectra of the samples exhibiting the peaks at their respective binding energies for C1s, O1s, Pt4f and Ti2p. The high resolution Pt4f peak of the Photo-Pt-graphite-TiO_2_ consisted of three individual peaks at 71.2, 72.4 and 73.7 eV corresponding to Pt^0^, Pt^II^ and Pt^IV^ oxidation states (Fig. 2C). The Pt^0^ was found to be the predominant oxidation state with 65.5% followed by 27.3% of Pt^II^ and 7.2% of Pt^IV^. Figure 2D shows the decomposition of Pt 4 f peak for Photo-Pt-CNT-TiO_2_. This too consists of three peaks at 71.4, 72.0, and 73.5 corresponding to Pt^0^, Pt^II^ and Pt^IV^ respectively. Similar to graphite based material Photo-Pt-CNT-TiO_2_ also consists predominantly Pt^0^ with around 62.6% along with small proportions of Pt^II^ (17.6%) and Pt^IV^(19.8%). The shift of Pt4f peak in the case of Photo-Pt-CNT-TiO_2_ can be ascribed to strong metal substrate interaction. Corroborating the results from TEM and XPS, it can be understood that no/less Pt-nps on GO and different particle sizes on different substrates are all based on the electronic conductivity of the carbon allotropes. Electronic conductivity in graphite, CNT and graphene are solely due to the continuous π electron cloud. When this π electron cloud is interrupted by some oxygen containing functional groups, the electronic conductivity gets hindered. Hence, the presence of various oxygen functional groups on the surface of graphene oxide makes it insulating in nature. Whereas, in case of graphite and CNT, the conductivity is higher as these materials have been used without any functionalization. The diffusion length of the photoelectron depends on the electronic conductivity of the substrate used. CNT is a better electronic conductor than graphite. This enables better conduction of photoelectrons with which Pt precursor is reduced all over the surface of CNT with many nucleation sites to generate small and well distributed Pt-nps. In the same line, CNT being better electronic conductor than that of graphite, smaller and more uniform Pt-np distribution resulted as expected. On the other hand, in case of GO, covalently bonded oxygen functionalities are readily reduced than that of adsorbed reducing species in the presence of photo-electron^27^ resulting in reduced graphene oxide (RGO) with fewer or no Pt particles. The cyclic voltammetry (CV) and linear sweep voltammetry using rotating disc electrode studies for evaluating the ORR behavior was benchmarked with commercial TEC10E50E. Cyclic voltammograms given in Fig. 3A showed a typical Pt-carbon type fingerprint peaks for H_2_ adsorption and desorption at 0.05 > E < 0.3 V. The electrochemical active surface area (ECSA) of the materials (see ESI for formula) was determined. Figure 3B shows the ECSA of all the electrocatalysts mentioned above. ECSA of the materials was found to be in the following range, Photo-Pt-CNT-TiO_2_ (74.13 m^2^/g), Photo-Pt-Graphite-TiO_2_ (53.08 m^2^/g). Highest ECSA of Photo-Pt-CNT-TiO_2_ can be attributed to the smallest particle size (1 ± 0.3 nm) of other 2 materials. Pt-nps in these materials can be seen as two types, the ones present on the TiO_2_ and the other on the surface of carbon. In Photo-Pt-TiO_2_-GO the majority of Pt-nps are present only on the surface of TiO_2_ and less/no Pt-nps on GO. With 1.6 wt% of Pt Photo-Pt-GO-TiO_2_ showed less ECSA of 11 m^2^g^−1^. This shows that the Pt on TiO_2_ is active for ORR but not as efficient as Pt on conducting carbon. The week catalytic activity can be ascribed to moderate electrochemical interaction between Pt-nps and semiconductor^28^,^29^.

**Figure 4:**
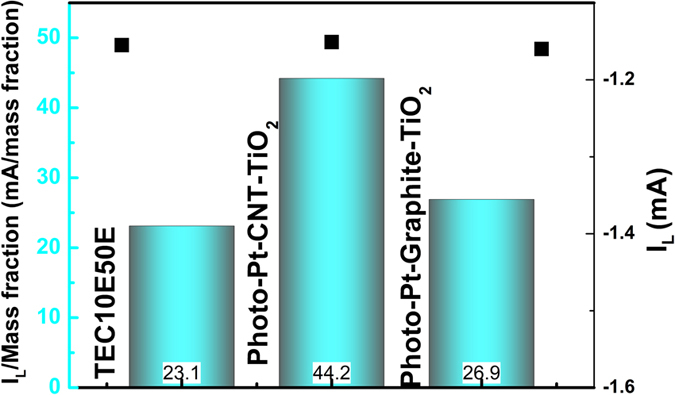
Bar-graph showing limiting current and normalized IL with mass fraction.

Further to understand the ORR kinetics, RDE measurements were carried out. Figure 3C compares the polarization curves of both the indigenous materials. Both Photo-Pt-CNT-TiO_2_ and Photo-Pt-graphite-TiO_2_ exhibited high ORR activity with the onset potential of 0.93 V and half wave potential of 0.85 V. A marginal photo generated catalysts compared to commercial catalyst (Figure ESI 4E) can be due to the decrease in the electronic conductivity of the sample by containing TiO_2_. But, this can be trade-off parameter compared to the amount of Pt loaded. The diffusion limiting current for all the samples was obtained at a potential region E < 0.6 V. Rotation dependent polarization curves were recorded to evaluate the kinetic parameters (Figure ESI 4) of ORR. The number of electrons transfer during the ORR calculated employing Koutecky–Levich (K–L) plots (Figure ESI 4) was found to be ~4 at 0.8–0.9 V. This corroborates that the ORR is a single step 4 electron process and not sequential 2 electrons process. The mass activity (MA) and specific activity (SA) based on the normalization of Pt loading and it’s ECSA respectively have been evaluated (Fig. 3D). Interestingly, though the ECSA of the home-made materials were slightly lesser than that of the commercial reference, MA of all the samples were found to be same (around 350 A/g) and SA was found to be in the order of Pt-Graphite-TiO_2_ (6.6 A/m^2^) >Pt-CNT-TiO_2_ (4.8 A/m^2^) >TEC10E50E (4.6 A/m^2^). The high SA of Pt-Graphite-TiO_2_ can be ascribed to low ECSA and larger particle size. The limiting current (I_L_) normalized with mass fraction of platinum on working electrode was another figure of merit to appreciate the ORR catalytic activity of the photo-generated catalysts with low Pt content (Fig. 4). In Fig. 4, high limiting current of the materials under study was equal to that of commercial catalyst and this high current normalized with low mass fraction of Pt to the corresponding sample showed high capacity. This showed a 2 times higher activity in case of CNT based material and a marginal increase in case of graphite based compared to the reference material. Here it would be worthy to emphasize that photo generated Pt materials contain approximately ~15 times less Pt in Photo-Pt-graphite-TiO_2_ and ~20 times in the case of Photo-Pt-CNT-TiO_2_ than that of reference material (TEC10E50E) with 50 wt% of Pt. This clearly demonstrates that the home-materials are highly active. The high performance in these hybrid material can be ascribed to, i) uniform distribution of Pt nano particles resulting in high ECSA, enabling easy diffusion of oxygen on Pt, ii) strong metal substrate interaction (SMSI) i.e., strong anchoring of Pt to carbon will lead to efficient electron transfer to reactant and, iii) moderate strength of Pt-oxygen coordination (because of SMSI) allows easy desorption of intermediates and the final product viz., H_2_O. These reasons can be attributed to efficient catalytic reaction as seen in this work.

## Conclusions

In conclusion, a highly reproducible, environmentally benign and ultrafast method was developed for the preparation of active ORR catalyst (for use in fuel cell and lithium-air battery). The method uses solar light as the only source of energy in the preparation of this catalytically efficient composite utilizing abundant and environmentally benign TiO_2_ as photo-catalyst. Using this green methodology, preparation of novel composites containing TiO_2_ particles, CNT/Graphite substrates and platinum nanoparticles was successfully carried out. As prepared material showed excellent ORR activity comparable to the best of commercial ORR catalysts. Both materials made showed comparable MA and higher SA than that of commercial one. It can also be confirmed that this method is feasible for all the conducting carbon substrates. The above results clearly demonstrates that the novel, green and a very viable method of Pt decoration method is as efficient as rather more efficient as that of well-established methods using SRA.

## Methods

Graphite, TiO_2_ and chloroplatinic acid were purchased from Sigma Aldrich. Graphene oxide (GO) and CNT were prepared by modified Hummers method and CVD process respectively (see ESI for procedure).

For the photo-reduction process, graphite, GO or CNTs weighing 90 mg was ultrasonicated in deionized water for nearly 2 hrs to obtain a uniform dispersion. To this uniform dispersion, 10 mg (10 wt%) of TiO_2_ (anatase commercially obtained from Sigma Aldrich with the particle size of 50–60 nm) was introduced and ultrasonicated for 15 min. 800 μL of 0.045 M aq. chloroplatinic acid solution was added to this mixture and was irradiated by a simulated solar light (Peccel, PEC-L15 solar simulator) with a maximum of 1 sun intensity (100 mWcm^−1^) for 5 hrs under constant stirring. The material was filtered, washed with deionized water and dried under vacuum overnight at room temperature. To determine the exact amount of Pt present on all the three catalysts (Photo-Pt-graphite-TiO_2_, Photo-Pt-GO-TiO_2_ and Photo-Pt-CNT-TiO_2_), inductively coupled plasma mass spectrometry (ICP-MS) measurements were done using Shimadzu ICPE-9000 (see ESI for procedure). The material was then subjected to morphological characterization and elemental analysis was carried out by using transmission electron microscopy (TEM) on Hitachi H-7650 model and X-ray photoelectron spectroscopy (XPS) on S-probe TM 2803 instrument.

Further, the material was studied for its electro catalytic activity by observing the ORR behaviour using cyclic voltammetry (CV) and rotating disc electrode (RDE) measurements (Automatic polarization system HZ-5000, Dynamic electrode HR-301 and Dynamic electrode controller HR-502). These electrochemical studies were performed using conventional three electrode system; electrocatalyst coated on to glassy carbon electrode as working electrode, platinum wire as counter and reversible hydrogen electrode (RHE) was used as reference electrode at 30 °C in 0.1 M HClO_4_ aq. The Pt loading on the electrodes made of home-made materials were maintained at 5.07 μg/cm^2^ and that of reference material TEC10E50E (received from Tanaka Kikinzoku Kogyo K.K., contains ~50 wt% of Pt on high surface area carbon) at 20.26 μg/cm^2^. CV was measured by sweeping the voltage between 0.05 V–1.20 V at the sweep rate of 50 mVs^−1^ in nitrogen saturated 0.1 M HClO_4_ aq. at 30 °C. Linear sweep voltammetry was studied using RDE technique across 0.05–1.00 V with the voltage sweep rate of 20 mVs^−1^ in oxygen saturated 0.1 M HClO_4_ aq. solution. The rate of rotation in RDE was varied from 400–3600 rpm.

## Additional Information

**How to cite this article**: Rajashekar, B. *et al*. Sacrificial Reducing Agent Free Photo-Generation of Platinum Nano Particle over Carbon/TiO_2_ for Highly Efficient Oxygen Reduction Reaction. *Sci. Rep.* **6**, 37006; doi: 10.1038/srep37006 (2016).

**Publisher’s note**: Springer Nature remains neutral with regard to jurisdictional claims in published maps and institutional affiliations.

## Supplementary Material

Supplementary Information
