# Supplementary material for: Sacrificial Reducing Agent Free Photo-Generation of Platinum Nano Particle over Carbon/TiO2 for Highly Efficient Oxygen Reduction Reaction
Source: Sci Rep. 2016 Nov 15;6:37006. doi: 10.1038/srep37006 (PMC5109473; doi:10.1038/srep37006)
Supplement: Supplementary Information [file srep37006-s1.doc]

**Electronic Supplementary Information**

**Sacrificial Reducing Agent Free Photo-Generation of Platinum Nano Particle over Carbon/TiO_2_ for Highly Efficient Oxygen Reduction Reaction**

**Badam Rajashekar^1^, Raman Vedarajan^1^, Kazuki Okaya^2^, Koichi Matsutani^2^, Noriyoshi Matsumi^1^**

**^1^School of Materials Science, Japan Advanced Institute of Science and Technology, 1-1 Asahidai, Nomi, Ishikawa 923-1292, Japan**

**^2^Tanaka Kikinzoku Kogyo K.K, Nagatoro 2-14, Hiratsuka, Kanagawa 254-0021, Japan**

**Preparation of GO by modified Hummers method**

GO was prepared using modified Hummers methods. Graphite (1 g) was stirred in 23 mL of concentrated H_2_SO_4_ for 24 h. Then, 3 g of KMnO_4_ was slowly added while keeping the round-bottom flask in an ice bath. Subsequently, the mixture was stirred at 35–40 ^o^C for 30 min, then at 65–80 ^o^C for 45 min. Next, 46 mL of water was added into the mixture and heated at 98–105 ^o^C for 30 min. The reaction was terminated by addition of distilled water (140 mL) and 30% H_2_O_2_ solution (10 mL), causing the color of the suspension to turn bright yellow. The products were washed with 5% HClaq. and then with deionized water by repeated centrifugation. Finally, the mixture was transferred to a culture dish and dried at 90 ^o^C.

**Synthesis of carbon nanotubes (MmNi3)**

CNTs were synthesized using a single stage furnace thermal CVD^1^ facility by catalytic decomposition of acetylene over misch metal (Mm) based AB3 alloy hydride catalyst. These catalysts were prepared through hydrogen decrepitation route by performing several cycles of hydrogenation/dehydrogenation of the alloy using a Sieverts apparatus. Fine powders of alloy obtained after several cycles of hydrogenation/dehydrogenation were directly placed in a quartz boat and kept at the center of a quartz tube, which was placed inside a tubular furnace. Hydrogen (50 sccm) was introduced into the quartz tube for 30 min at 500 °C, and then furnace was heated up to 700 °C, followed by the introduction of acetylene for 30 min with a flow rate of 50 sccm. Acetylene flow was stopped, and the furnace was cooled to room temperature. Argon flow was maintained throughout the experiment.

**Inductively Coupled Plasma Mass Spectrometry**

To determine the exact amount of Pt present on all the four catalysts, inductively coupled plasma mass spectrometry (ICP-MS) measurements were done. In a typical experimental procedure, 20 mg of each catalyst was weighed and heated at 150 ^o^C for 30 min under N_2_(0.2 Lmin^-1^):H_2_(1.0 Lmin^-1^). The mixture was then calcinated for 60 mins at 800 ^o^C in air. The cooled residue was dissolved in 5 mL of aquaregia. The obtained solution was filtered and the filtrate was diluted with water and made up to 100 mL. This solution was used in ICP experiment using Shimadzu ICPE-9000.

**TEM micrographs of Photo-Pt-GO-TiO_2_**


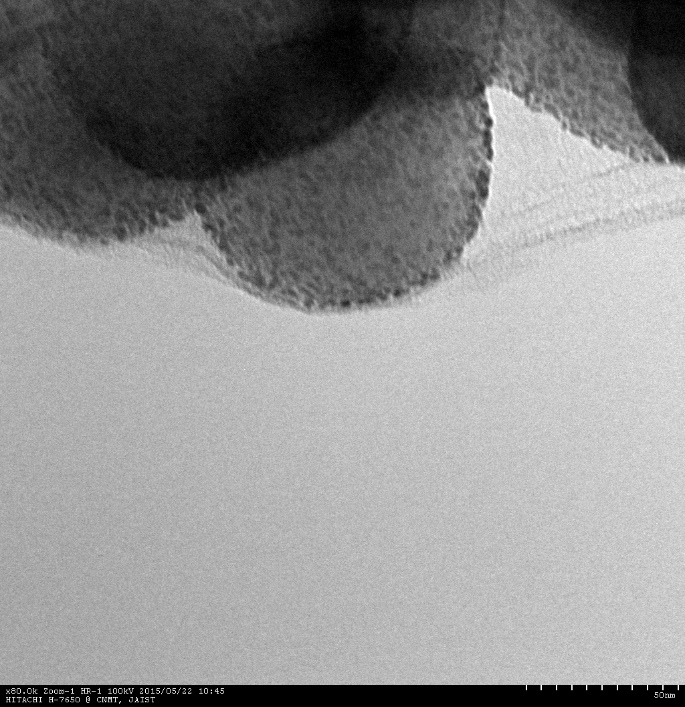

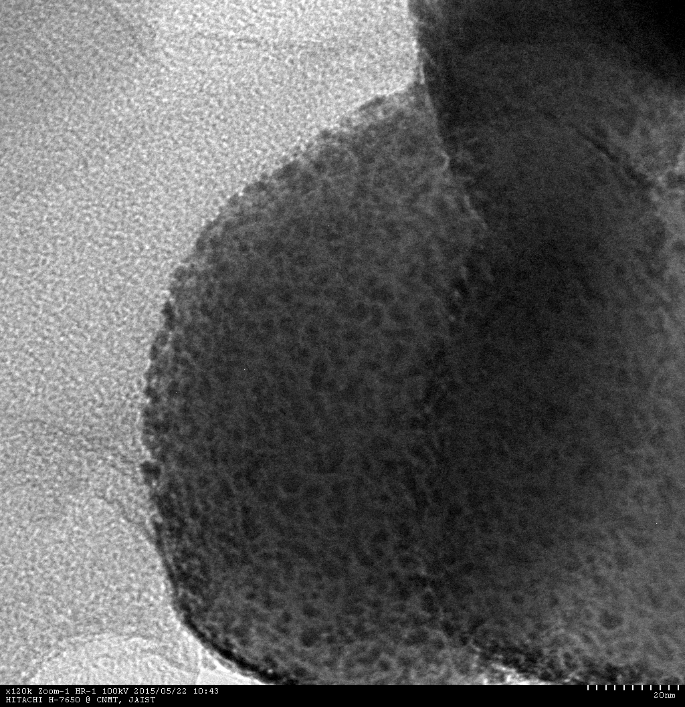


**50 nm**

**20 nm**

*Figure ESI 1 TEM images of Photo-Pt-GO-TiO_2_ (A-B)*

**A**

**B**

**Histograms for Figure 1A and Figure 1C of main text**

**
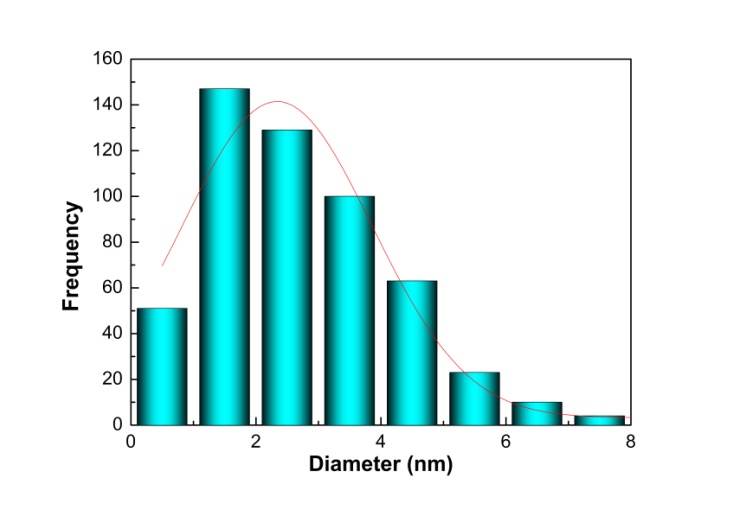

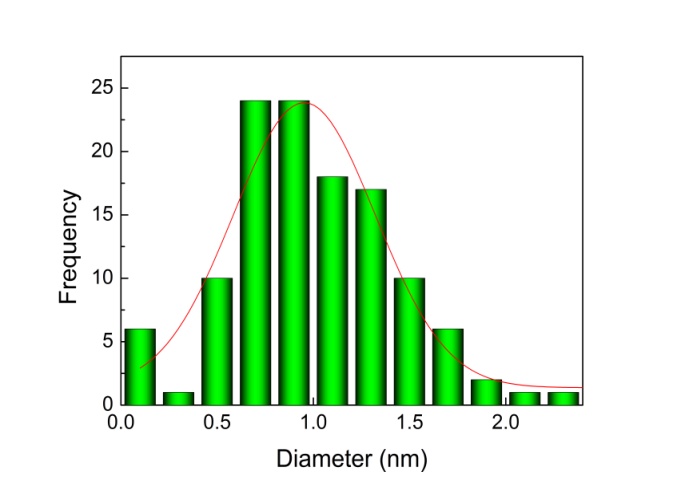
**

**A**

**B**

*Figure ESI 2 Histograms showing average particle size of Photo-Pt-Graphite-TiO_2_ (1) and Photo-Pt-CNT-TiO_2_ (B)*

**Electrocatalytic ORR activity and Electrochemical Surface Area (ECSA) calculation:**

The following is the equation with which the ECSA was calculated.

Charge [Q_H_μC/cm^2^]

ECSA =

Q_m_ X Pt loading [g/cm^2^]

Where Q_H_ is the charge corresponding to the hydrogen desorption peak, Q_M_ = 210 μC/cm^2^ is the electrical charge associated with the monolayer adsorption of hydrogen on Pt and Pt loading is the amount of Pt loaded onto the electrode.


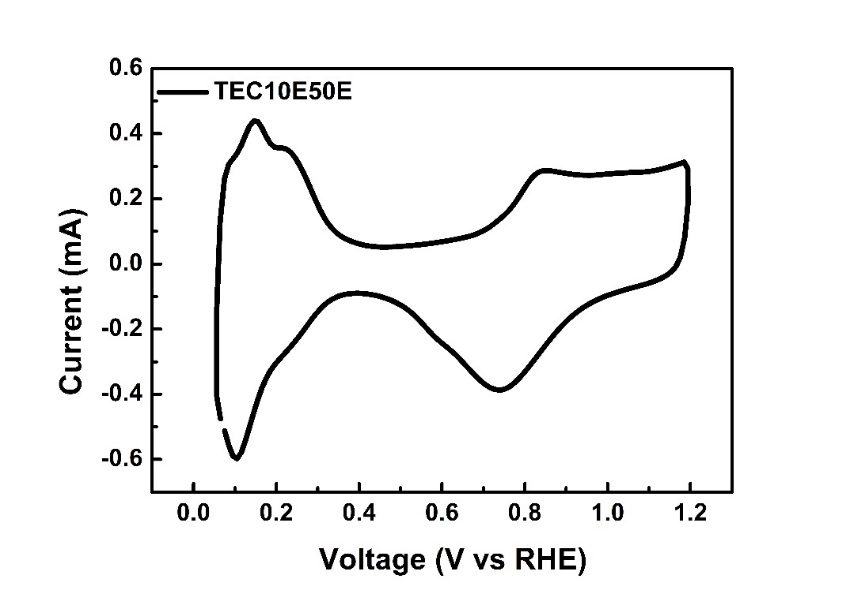

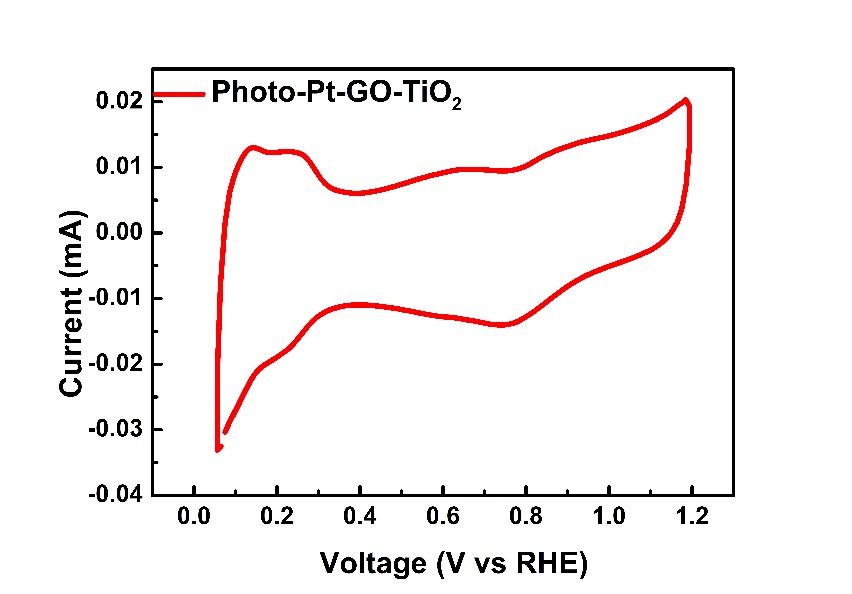


*Figure ESI 3. Cyclic voltammograms of TEC10E50E and Photo-Pt-GO-TiO_2_.*

**Pt loading on GC-20.3μg/cm^2^**

**Pt loading on GC-5.06μg/cm^2^**


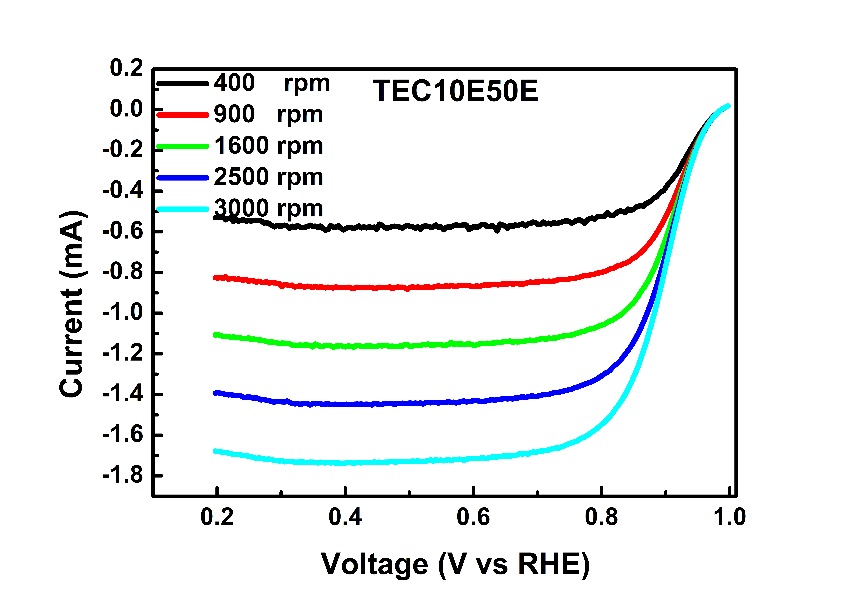

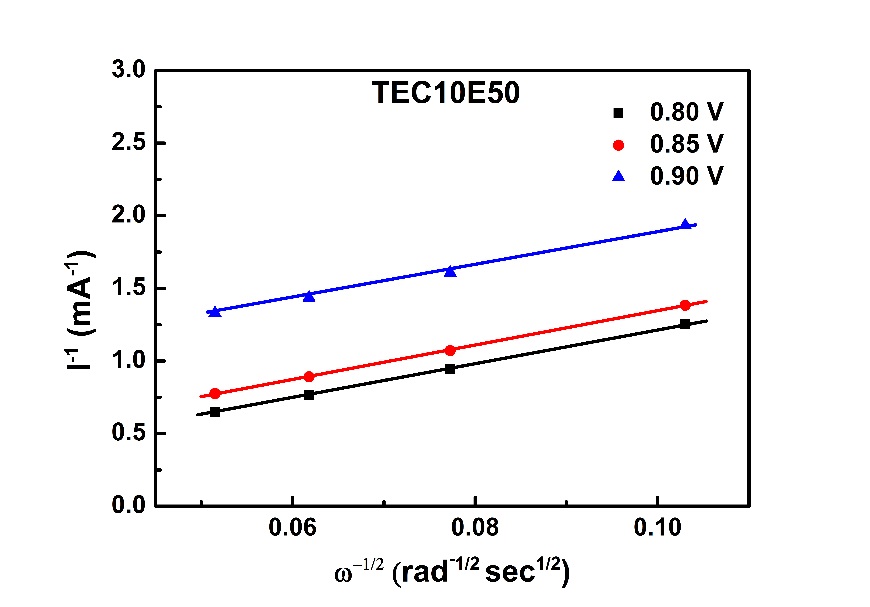


*Figure ESI 4. RDE curves for Photo-Pt-Graphite-TiO_2_ (A) Photo-Pt-CNT-TiO_2_ (C)and TEC10E50E (E) at 30 ^o^C in oxygen saturated 0.1 M HClO_4_ at scan rate of 20 mV/s at different rotation speeds (400, 900, 1600, 2500, 3600 rpm) and respective Koutecky-Levich plots (B, D and F) at 0.80, 0.85 and 0.90 V vs RHE.*


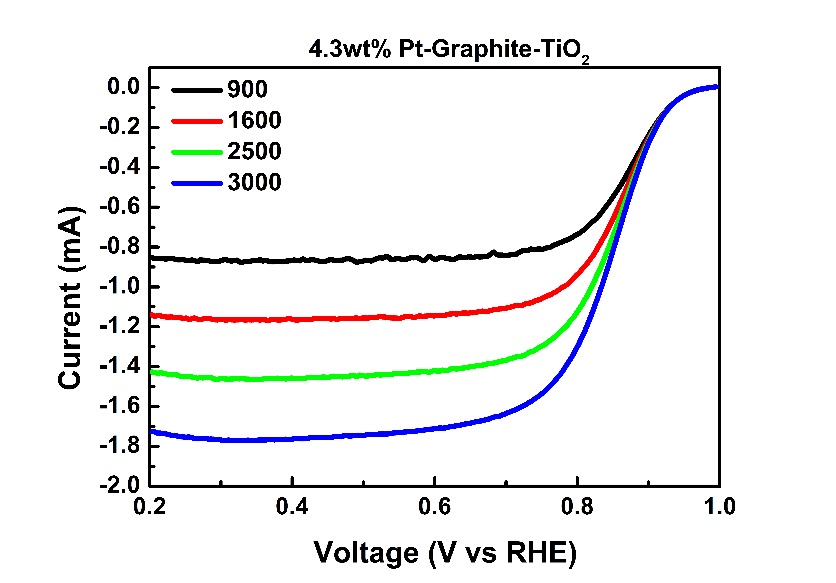

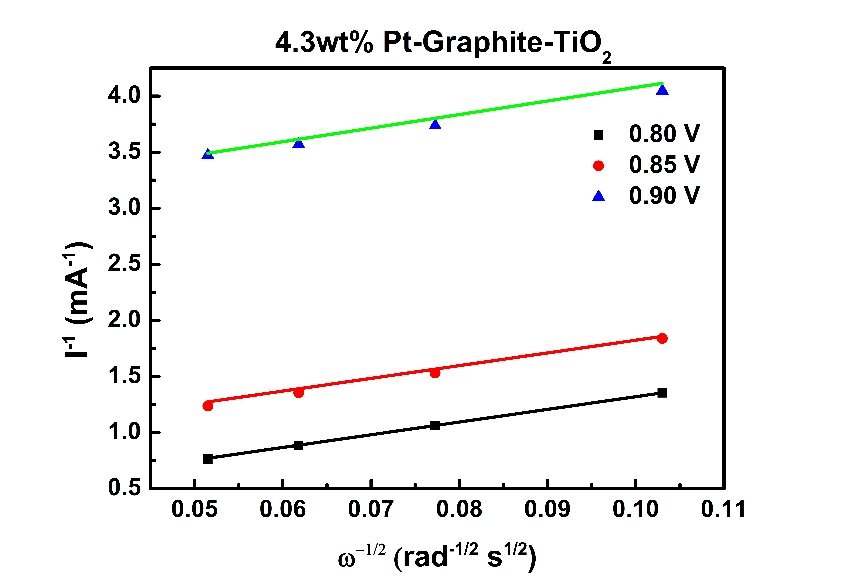

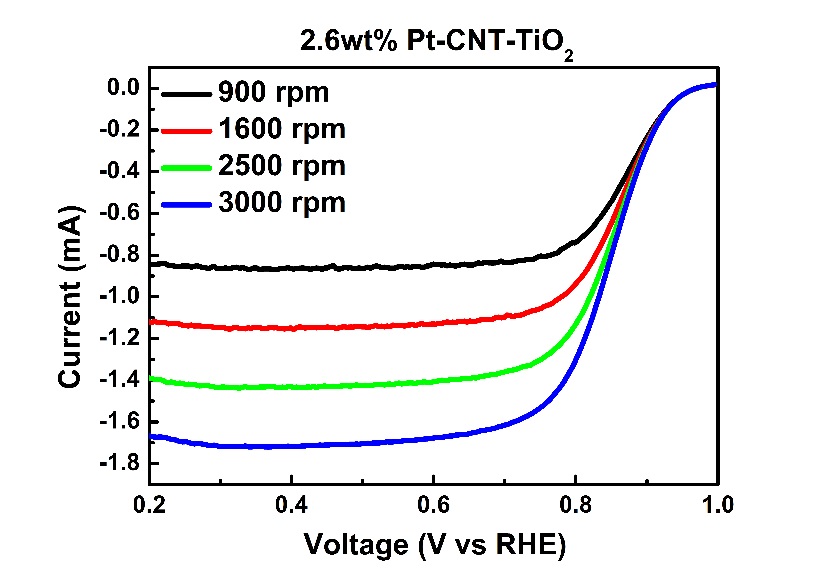

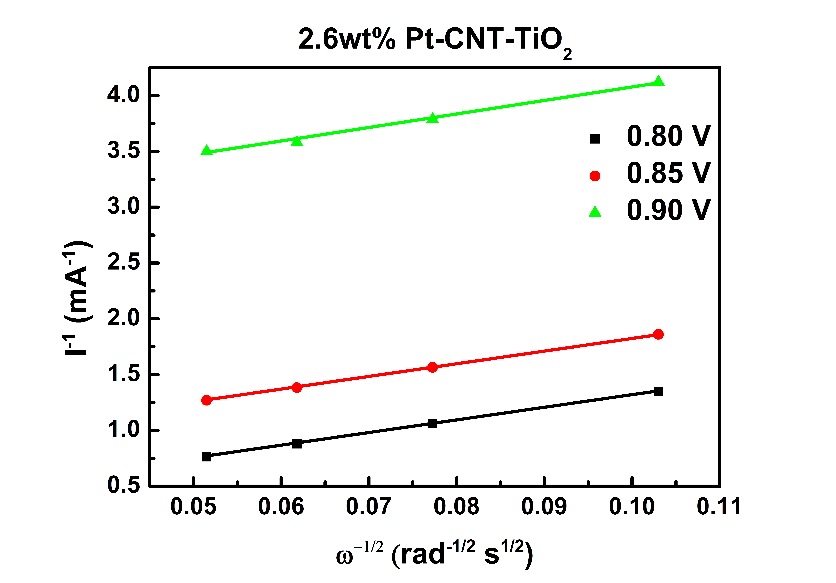


**A**

**B**

**C**

**D**

**E**

**F**

Reference:

1. Eswaraiah, V., Aravind, S. S. J. & Ramaprabhu, S. Facile synthesis of one dimensional graphene wrapped carbon nanotube composites by chemical vapour deposition. *J. Mater. Chem.***21,** 15179–15182 (2011).
